# Letter from Berlin

**Published:** 1878-03

**Authors:** Chas. F. A. Nichell

**Affiliations:** 105 Friederich St., Berlin


### Correspondence.

105 Friederich St.,
BERLIN, Jan'y 27, 1878.

*Editor of the Buffalo Medical and Surgical Journal:*

MY DEAR DOCTOR:—A longer time has elapsed since my arrival in this city than I intended before writing to you, but I delayed from day to day in the hope of writing something interesting to you and your Journal. Were you a lady interested in marriage questions I could fill you brim full with the coming wedding of the Princesses Charlotte and Elizabeth, or were you a hunter after decorations I could tell you a good deal about the Decoration feast just held, but as you are a man, who has no eye or ear for such things I must try and tell you what is being done here in medicine and how it is done, trusting that you will not consider me as a thief upon your time.

While the remark that you made to me just before leaving Buffalo last fall is quite true, viz., "that the European physicians did not show any superiority over the American and that we could show as good men as any country." we must admit that such is only the case with our better men and that our general practitioners are not so well qualified for their work as those of Germany. The preliminary education and the thorough didactic and clinical training which the men receive here is far superior to that which is given to our students and the consequences are those already stated, viz., better general practitioners. The fault with us does not lie so much in the character of the didactic teaching as its short duration and great hurriedness—nor is there a want of clinical material, but the proper use of it, by making the student step forward make his diagnosis and pursue his treatment under the guidance of competent supervision. If the hurry to make doctors which some of our schools of medicine exhibit could be checked and in the future only the quality of the men turned out be considered it would be a great blessing. But by far the greatest blessing to the profession would be to require, as in this country, a State Examination. The degree of Doctor of Medicine conferred here does not permit the so-dubbed iudividual to practice medicine but he must first pass a state examination which lasts from four to six months. Having passed such an examination the physician is protected by government in his interests and is not the "Doc." of every woodsawyer or cobbler.

While in the specialty which I have chosen as the future field of my activity, the United States can show as proud a record as any country and perhaps grander achievements in the operative department than any other nation, yet the opportunities for studying gynæcology are so few that it has paid me to come here in the pursuit of this department of medicine. As yet the greater part of the American people would be shocked at the idea of bringing woman after woman upon the examination table and before a class of 60 to 80 medical men and I cannot but look back with a smile when I think how Prof. White marched a few members of the graduating class into a room, where a patient lay covered so that her modesty might not be hurt, to enable them to listen to the fœtal heart. Nor can I sufficiently condemn the course of the profession when he attempted to give students a better opportunity of clinical study, thereby depriving them of instruction most valuable in their earlier career and which might perhaps have saved them great annoyances and the patient great suffering, perhaps injury.

Speaking of my opportunities of studying gynæcology, my good star led me to Dr. A. Martin, son of the late Prof. E. Martin, and Lecturer of gynæcology of the University of Berlin, whose clinical assistant I have become. At the free clinic we have from 30 to 40 patients per day. The gentlemen present (all graduates) take turns by twos and threes in examining the patients, making their diagnosis, etc., and applying the treatment. Besides the examinations on part of the Doctors I examine each case individually and assume under Martin a general direction of the patients. Often I run the clinic through all alone. The character of cases presenting themselves cover the whole field of gynæcology, from a chronic metritis to the uterine fibroid, or ovarian tumor. All such cases requiring operative interference are sent to a private clinic, to which also cases out of private practice are directed, so that a large amount of material is constantly at hand. This private clinic is in my charge and a good opportunity is thereby offered me to follow the results of operations which might otherwise be lost to my view. During the month of Nov. '77, ten abdominal sections were made, viz, two castrations, for intersticial and subservious fibroids of the womb, to produce premature involution, one extra-uterine fœtation, two ovariotomies duplex and five ovariotomies; the tumors being some multi some unilocular. All patients recovered, some being discharged in fourteen days. In one case of double ovariotomy the tumor was enucleated as suggested by yourself out of the broad ligament with great satisfaction, the procedure leading in all probablity to the success of the operation. One case of ovariotomy per vagina was very happy in its result. During the past two and one half months we have made twenty-two amputations of the womb (vaginal portion), two Emmet's operations for laceration of the cervix, two vesico vaginal fistulæ (unsuccessful), one kolporraphy, some fifteen stenosis os uteri ,etc., etc., so that you will observe material is not in the least wanting. Perhaps it may not be out of character here to refer to the bi-manual or combined examination over the simple digital as generally practiced. By this method the examiner stands in front of the patient, and while making the vaginal exploration with the right or left hand he applies the other upon the abdomen and by steady but gentle pressure brings the organs of the pelvis between the fingers, thereby obtaining a clearer idea of their size, consistency, position and relation. This method although adopted by some at home, is yet according to my observation too limited in its application. Its advantages are such that I doubt whether any physician will content himself with a simple digital exploration after having accustomed himself to the combined method.

As your acquaintance with the Med. Literature of the United States is such as to entitle your judgment to great weight and consideration, Iwould like to ask you how a work upon the Versions and Flexions of the Uterus, by E. Martin, late Professor of Obstetrics and Gynæcology would likely be received. The work is based upon some 850 cases, which have been studied with special reference to cure and not simply relieve. Prof. White was probably personally acquainted with the gentleman, as at the time of his visit he must have held the chair at Wurzburg, being succeeded there by Scanzoni. I have the work pretty well along in its translation, and if it is likely to meet a favorable reception or supply any demand, I should be glad to push the matter and bespeak your interest. The book would occupy about two hundred pages. Please let me know your views on this subject, and if it would not be asking too much, also those of Prof. White.

Besides following my special department I occasionally visit Langenbeck, Virchow, French's, etc. Langenbeck has a daily clinic from two till five, during which he operates and demonstrates. He has a list of all students having put their names down and calls upon them to step forward and diagnose the case under consideration. After the diagnosis is established the gentleman stays and becomes the principal assistant during that operation. As soon as the operation is done the next patient is presented and another student is called. After Langenbeck gets done (5 P. M.,) his assistant continues the clinic until 7 P. M. Prof. Virchow begins his lecture at 8.15 A. M. and demonstrates until 11 A. M. Medical lectures begin at 7 A. M. and last until 10 P. M., and not only week days but also Sundays. Of course the students cannot attend all, so they select the lectures they wish to hear and paddle along whither their own sweet will leads them. The attendance of Americans is quite respectable this season, Boston, New York and Philadelphia being especially represented. Besides myself there are two other Californians here. Having probably exhausted your patience completely, permit me to come to a close with the hope of soon hearing from you, and permit me to subscribe myself

Ever Yours,

CHAS. F. A. NICHELL.

